# Optimization and Real-World Implementation of Guideline-Directed Medical Therapy in Heart Failure With Reduced Ejection Fraction: A Contemporary Clinical Review

**DOI:** 10.14740/cr2195

**Published:** 2026-04-15

**Authors:** Ehsan Shahverdi, Amin Shahverdi, Carsten Schneider, Mathias Lange, Lars Roman Herda

**Affiliations:** a Department of Cardiology, Rhythmology, Angiology and Intensive Care Medicine, Heart Center Osnabrück, Hospital Osnabrueck, Westphalian Wilhelms University of Muenster, Osnabrueck, Germany; b Department of Emergency Medicine, Clemens Hospital, Muenster, Germany

**Keywords:** Heart failure with reduced ejection fraction, Guideline-directed medical therapy, GDMT implementation, Rapid initiation, Therapeutic sequencing, Advanced heart failure

## Abstract

Heart failure with reduced ejection fraction (HFrEF) remains a major global health burden despite the availability of highly effective disease-modifying therapies. Contemporary guidelines consistently endorse four foundational pillars of guideline-directed medical therapy (GDMT); however, real-world implementation remains incomplete, with delayed initiation, suboptimal sequencing, and low rates of target dose achievement. This review aims to provide a clinically oriented, implementation-focused framework for optimizing GDMT in HFrEF, emphasizing early initiation, practical sequencing strategies, structured up-titration, and phenotype-guided prioritization in routine practice. A narrative synthesis of major randomized clinical trials, contemporary guideline recommendations, and real-world registry data was performed. Evidence was integrated to develop pragmatic strategies for rapid initiation (“four drugs in 4 weeks”), safe titration, and overcoming common barriers to implementation. Early and comprehensive initiation of renin–angiotensin system inhibition (preferably with angiotensin receptor–neprilysin inhibitors), evidence-based beta-blockers, mineralocorticoid receptor antagonists, and sodium–glucose cotransporter 2 (SGLT2) inhibitors is associated with rapid and sustained reductions in hospitalization and mortality. Clinical benefits of SGLT2 inhibitors emerge within weeks of initiation, reinforcing the importance of early deployment. Nevertheless, substantial residual risk persists even under quadruple therapy, and real-world data demonstrate persistent underutilization of foundational therapies. Structured follow-up, phenotype-guided prioritization, and protocol-driven titration strategies may facilitate safe and comprehensive implementation. Adjunctive device therapy, timely referral for advanced heart failure evaluation, and integration of cardiac rehabilitation further enhance long-term outcomes. The contemporary challenge in HFrEF management lies not only in identifying effective therapies, but in ensuring their timely and coordinated implementation. An implementation-oriented approach that prioritizes rapid initiation, structured up-titration, and individualized clinical decision-making may help bridge the gap between guideline recommendations and real-world practice.

## Introduction

Heart failure with reduced ejection fraction (HFrEF) remains a major global health challenge and is associated with substantial morbidity, mortality, and healthcare utilization despite major advances in cardiovascular therapeutics [1, 2]. Over the past three decades, randomized clinical trials have established a robust evidence base demonstrating that neurohormonal modulation and, more recently, sodium–glucose cotransporter 2 (SGLT2) inhibition significantly reduce hospitalization and mortality in HFrEF [3–5]. These findings have been incorporated into contemporary clinical practice guidelines issued by the European Society of Cardiology and the American Heart Association, American College of Cardiology, and Heart Failure Society of America [6, 7].

Current guidelines clearly define four foundational pillars of guideline-directed medical therapy (GDMT)—renin–angiotensin system inhibition (preferably with angiotensin receptor–neprilysin inhibitors), evidence-based beta-blockers, mineralocorticoid receptor antagonists, and SGLT2 inhibitors—as the cornerstone of contemporary HFrEF management. However, despite strong recommendations and high-level evidence, real-world implementation of comprehensive GDMT remains inconsistent. Registry data demonstrate persistent underutilization of foundational therapies, delayed initiation, and low rates of achievement of target or maximally tolerated doses [8, 9].

Importantly, the majority of excess risk in HFrEF occurs early after diagnosis or hospitalization, a period during which therapeutic inertia, concerns regarding tolerability, and fragmented follow-up may delay comprehensive treatment initiation [10]. As a result, the challenge in modern heart failure care is no longer merely identifying effective therapies, but ensuring their timely, coordinated, and sustained implementation across diverse patient populations.

This review is not intended to replicate existing guideline documents, but rather to provide a clinically oriented, implementation-focused framework for the optimization of GDMT in HFrEF. Emphasis is placed on early initiation strategies, sequencing paradigms such as “four drugs in 4 weeks,” structured up-titration, phenotype-guided prioritization, and practical solutions to common barriers encountered in routine practice. In addition, contemporary controversies—including management in elderly and frail populations, heart failure with improved ejection fraction (HFimpEF), sex-based differences, residual risk despite quadruple therapy, and considerations for advanced heart failure referral—are discussed to provide interpretive context beyond guideline summaries.

By integrating pathophysiologic insight, trial evidence, and real-world implementation challenges, this review aims to bridge the gap between consensus recommendations and practical clinical delivery of comprehensive HFrEF care.

## Pathophysiologic Basis of GDMT in HFrEF

HFrEF is characterized by progressive impairment of left ventricular systolic function accompanied by maladaptive neurohormonal activation. Reduced cardiac output leads to compensatory activation of the renin–angiotensin–aldosterone system (RAAS) and the sympathetic nervous system, initially preserving perfusion but ultimately accelerating myocardial dysfunction, adverse ventricular remodeling, and clinical deterioration [3].

Chronic activation of the RAAS promotes vasoconstriction, sodium and water retention, myocardial fibrosis, and ventricular hypertrophy, all of which contribute to progressive heart failure and increased mortality [6]. Similarly, sustained sympathetic nervous system activation results in tachycardia, increased myocardial oxygen demand, arrhythmogenesis, and direct cardiomyocyte toxicity [3]. These maladaptive mechanisms provide the pathophysiologic rationale for long-standing cornerstone therapies such as angiotensin-converting enzyme inhibitors, angiotensin receptor blockers, and evidence-based beta-blockers.

More recent advances in HFrEF management have expanded the neurohormonal paradigm. Neprilysin inhibition augments endogenous natriuretic peptides, enhancing vasodilation, natriuresis, and inhibition of myocardial fibrosis and hypertrophy. The superiority of angiotensin receptor–neprilysin inhibitors over conventional RAAS blockade underscores the importance of simultaneously suppressing harmful neurohormonal pathways while enhancing protective mechanisms [4].

In addition, SGLT2 inhibitors exert favorable effects on heart failure outcomes through mechanisms that extend beyond glycemic control. Proposed pathways include osmotic diuresis, improved myocardial energetics, reduction of preload and afterload, attenuation of inflammation, and favorable effects on renal function, collectively contributing to reduced heart failure hospitalization and mortality in patients with HFrEF [5].

Aldosterone excess plays a central role in myocardial fibrosis, vascular inflammation, and potassium wasting. Mineralocorticoid receptor antagonists mitigate these effects and provide incremental benefit when added to background RAAS inhibition and beta-blockade, further reinforcing the concept of comprehensive neurohormonal modulation [6].

Taken together, these pathophysiologic insights form the foundation of contemporary GDMT in HFrEF. Effective management requires not only the use of individual evidence-based agents but also their early initiation and combined application to counteract multiple maladaptive pathways driving disease progression [7, 10].

## Contemporary Mechanistic Perspectives

Beyond classical neurohormonal activation, contemporary models of HFrEF recognize the contribution of systemic inflammation and maladaptive immune activation to progressive ventricular remodeling [11]. Pro-inflammatory cytokines promote myocardial fibrosis, apoptosis, and adverse structural remodeling.

The cardio-renal-metabolic axis has emerged as an integrative paradigm linking heart failure progression with renal dysfunction, insulin resistance, and metabolic dysregulation [12]. Alterations in myocardial energetics—including impaired mitochondrial function and reduced ATP generation—contribute to diminished contractile reserve and exercise intolerance [13].

In addition, coronary microvascular dysfunction may exacerbate myocardial ischemia in the absence of epicardial coronary disease, further promoting adverse remodeling and systolic impairment [14]. These mechanistic insights broaden the pathophysiologic framework of HFrEF and provide rationale for therapies exerting pleiotropic systemic effects beyond traditional neurohormonal modulation.

## Foundational Therapies in HFrEF

Contemporary management of HFrEF is centered on the early initiation and optimization of four foundational classes of GDMT, each targeting distinct yet complementary pathophysiologic pathways. Robust evidence from randomized controlled trials has demonstrated that combined use of these agents significantly reduces mortality and heart failure–related hospitalizations compared with partial or delayed implementation [6, 7]. The four foundational classes of GDMT in HFrEF and their principal clinical benefits are summarized in Table 1.

**Table 1:** Foundational Guideline-Directed Medical Therapy in HFrEF

Drug class	Key agents	Main clinical benefit
ARNI/ACEi/ARB	Sacubitril/valsartan; enalapril; losartan	Reduced mortality, hospitalization, and adverse remodeling
Evidence-based β-blockers	Carvedilol; metoprolol succinate; bisoprolol	Reduced mortality and sudden cardiac death; reverse remodeling
Mineralocorticoid receptor antagonists	Spironolactone; eplerenone	Reduced mortality and hospitalization; antifibrotic effects
SGLT2 inhibitors	Dapagliflozin; empagliflozin	Reduced HF hospitalization and cardiovascular death, irrespective of diabetes

ARNI: angiotensin receptor–neprilysin inhibitor; ACEi: angiotensin-converting enzyme inhibitor; ARB: angiotensin receptor blocker; SGLT2: sodium–glucose cotransporter 2; HF: heart failure; HFrEF: heart failure with reduced ejection fraction.

### Renin–angiotensin system inhibition and angiotensin receptor–neprilysin inhibitors

Inhibition of the renin–angiotensin system represents a cornerstone of HFrEF therapy. Angiotensin-converting enzyme inhibitors and angiotensin receptor blockers reduce afterload, mitigate adverse ventricular remodeling, and improve survival in patients with HFrEF [3]. However, angiotensin receptor–neprilysin inhibitors have demonstrated superior clinical outcomes compared with conventional RAAS blockade and are now recommended as first-line therapy in eligible patients [4, 6].

Early initiation of angiotensin receptor–neprilysin inhibitors is associated with rapid reductions in natriuretic peptide levels and improved clinical stability, supporting their use early in the disease course rather than as late substitution therapy [10]. Careful monitoring of blood pressure, renal function, and serum potassium is essential during initiation and titration.

### Evidence-based beta-blockers

Beta-blockers counteract chronic sympathetic nervous system activation, reduce heart rate, improve myocardial energetics, and reverse adverse ventricular remodeling. Large-scale clinical trials have consistently demonstrated mortality and morbidity benefits with specific agents, including carvedilol, metoprolol succinate, and bisoprolol, when used in patients with stable HFrEF [3].

Initiation of beta-blocker therapy should occur once patients are euvolemic and clinically stable, with gradual dose titration to target or maximally tolerated doses. Transient worsening of symptoms may occur during early titration; however, long-term benefits substantially outweigh short-term hemodynamic effects when therapy is appropriately managed [7, 10].

### Mineralocorticoid receptor antagonists

Mineralocorticoid receptor antagonists provide incremental benefit by attenuating aldosterone-mediated sodium retention, myocardial fibrosis, and vascular inflammation. Their addition to background RAAS inhibition and beta-blockade significantly reduces mortality and heart failure hospitalizations in patients with HFrEF [6].

Appropriate patient selection and vigilant monitoring are critical, particularly in individuals with impaired renal function or baseline hyperkalemia. When used judiciously, mineralocorticoid receptor antagonists represent an essential component of comprehensive neurohormonal blockade [9].

### SGLT2 inhibitors

SGLT2 inhibitors have emerged as a foundational therapy in HFrEF, demonstrating consistent reductions in heart failure hospitalization and cardiovascular mortality irrespective of diabetes status [5]. Their rapid onset of benefit, favorable safety profile, and minimal impact on blood pressure make them particularly attractive for early incorporation into treatment regimens.

Recent randomized trial evidence indicates that the clinical benefits of SGLT2 inhibitors emerge early, with separation of event curves for heart failure hospitalization observed within weeks of therapy initiation, supporting their prioritization during the earliest phase of GDMT implementation [5]. This early risk reduction is particularly relevant given the high vulnerability of patients following diagnosis or recent decompensation and reinforces the concept that timely initiation—rather than delayed sequencing—may meaningfully influence near-term outcomes.

Despite the substantial incremental benefit conferred by SGLT2 inhibitors and other foundational therapies, meaningful residual morbidity and mortality persist even among patients receiving comprehensive quadruple GDMT. This residual risk underscores the progressive nature of HFrEF and highlights the importance of sustained optimization, close follow-up, and consideration of adjunctive pharmacologic or device-based therapies when indicated [6, 7].

Importantly, contemporary registry data continue to demonstrate major implementation gaps, with underutilization of foundational therapies and low rates of achievement of target or maximally tolerated doses in real-world practice [8]. These observations emphasize that the clinical impact of SGLT2 inhibitors and other disease-modifying agents depends not only on efficacy in trials, but also on systematic implementation strategies that overcome therapeutic inertia.

Unlike traditional heart failure therapies, SGLT2 inhibitors require minimal dose titration and have a low risk of electrolyte disturbances or renal dysfunction when appropriately prescribed. Current guidelines therefore recommend their use as part of initial combination therapy in most patients with HFrEF [6, 7].

## Clinical Integration of Foundational Therapies

Growing evidence supports a strategy of rapid initiation of all four foundational therapies at low doses, followed by systematic up-titration, rather than sequential introduction of individual drug classes over prolonged periods [10]. This approach maximizes early clinical benefit, reduces the risk of therapeutic inertia, and improves long-term outcomes in patients with HFrEF.

## Role of Diuretics and Digoxin in HFrEF

While contemporary management of HFrEF prioritizes disease-modifying GDMT, symptomatic treatments remain essential components of comprehensive care.

Loop diuretics are recommended for the relief of congestion in patients with signs and symptoms of volume overload. Although diuretics have not been shown to improve survival, they provide rapid symptomatic improvement and reduce pulmonary and systemic congestion. Clinical trial data support their central role in decongestion strategies, particularly in acute decompensated heart failure [15]. Careful dose adjustment is required to avoid excessive volume depletion, renal dysfunction, and electrolyte disturbances.

Digoxin may be considered in selected patients with persistent symptoms despite optimized GDMT, particularly in those with concomitant atrial fibrillation requiring additional rate control. The Digitalis Investigation Group trial demonstrated a reduction in heart failure–related hospitalizations without a significant effect on overall mortality [16]. Given its narrow therapeutic index, close monitoring of serum levels and renal function is necessary, especially in elderly patients and those with impaired renal function.

## Sequencing and Rapid Initiation of GDMT

Traditional approaches to the management of HFrEF have often relied on a stepwise and sequential introduction of individual drug classes over several months. Although historically pragmatic, this strategy may delay comprehensive neurohormonal blockade and prolong exposure to elevated risk during the early and vulnerable phase following diagnosis or clinical decompensation. Accumulating trial data and contemporary registry analyses increasingly support a paradigm shift toward earlier and more comprehensive initiation of disease-modifying therapy [6, 7, 10].

Rather than prioritizing achievement of target doses for a single agent before introducing the next, current evidence favors early exposure to all four foundational classes of GDMT—renin–angiotensin system inhibition (preferably with angiotensin receptor–neprilysin inhibitors), evidence-based beta-blockers, mineralocorticoid receptor antagonists, and SGLT2 inhibitors. Early implementation has been associated with rapid and clinically meaningful reductions in heart failure hospitalization and cardiovascular mortality, with benefits emerging within weeks of therapy initiation [5].

A practical implementation framework that has gained increasing attention is the “four drugs in 4 weeks” strategy. This approach emphasizes initiation of all four foundational therapies within the first month following diagnosis or clinical stabilization, followed by systematic up-titration to maximally tolerated doses. By prioritizing early exposure to each prognostically beneficial agent, this model seeks to minimize delays in risk reduction and reduce therapeutic inertia, which remains a major barrier in real-world practice.

## Principles of Rapid Sequencing

Rapid sequencing does not imply simultaneous high-dose initiation. Instead, it involves introducing each foundational therapy at a low starting dose in a clinically stable and euvolemic patient, followed by close follow-up and structured dose escalation. The majority of mortality benefit associated with these agents appears to derive from treatment initiation itself, even at submaximal doses, reinforcing the importance of early deployment rather than delayed optimization [10].

SGLT2 inhibitors can generally be initiated early due to their favorable safety profile, minimal hemodynamic effects, and lack of requirement for dose titration. Angiotensin receptor–neprilysin inhibitors should be introduced once blood pressure and renal function permit, with careful monitoring during dose escalation. Beta-blockers should be initiated in clinically stable, euvolemic patients, recognizing that transient worsening of symptoms may occur during early titration but should not prompt premature discontinuation in the absence of true intolerance. Mineralocorticoid receptor antagonists may be incorporated early in patients without significant hyperkalemia or advanced renal dysfunction.

## Clinical Considerations Influencing Sequencing

Although the overarching goal is early comprehensive therapy, individual patient phenotype should inform prioritization. In patients with borderline blood pressure, early initiation of SGLT2 inhibitors and mineralocorticoid receptor antagonists may be advantageous due to their relatively modest impact on systemic blood pressure. Conversely, in patients with elevated resting heart rate or concomitant atrial fibrillation, earlier introduction and cautious up-titration of beta-blockers may provide additional symptomatic and prognostic benefit. In individuals with chronic kidney disease, renin–angiotensin system inhibition and SGLT2 inhibition should not be withheld unnecessarily, but careful laboratory monitoring is essential.

**Figure 1:**
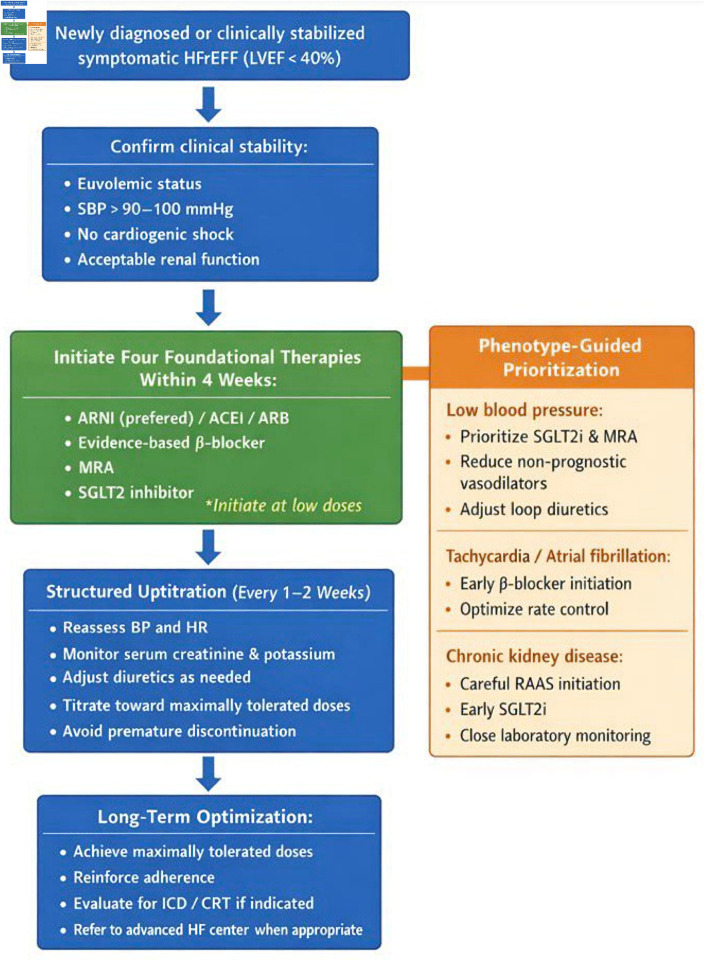
Practical phenotype-guided framework for rapid initiation and optimization of guideline-directed medical therapy (GDMT) in patients with heart failure with reduced ejection fraction (HFrEF). The algorithm emphasizes early initiation of all four foundational therapies (“four drugs in 4 weeks”) followed by structured up-titration and phenotype-specific prioritization. ARNI: angiotensin receptor–neprilysin inhibitor; ACEi: angiotensin-converting enzyme inhibitor; ARB: angiotensin receptor blocker; MRA: mineralocorticoid receptor antagonist; SGLT2i: sodium–glucose cotransporter 2 inhibitor; BP: blood pressure; HR: heart rate; ICD: implantable cardioverter-defibrillator; CRT: cardiac resynchronization therapy; LVEF: left ventricular ejection fraction; SBP: systolic blood pressure.

Importantly, rapid sequencing must be accompanied by structured follow-up, ideally within 1–2 weeks after initiation or dose adjustment. Regular assessment of blood pressure, heart rate, renal function, and serum electrolytes facilitates safe up-titration and reinforces adherence. A protocol-driven, multidisciplinary approach may further enhance successful implementation and reduce clinical inertia [7, 10]. A practical, phenotype-guided implementation framework integrating rapid initiation, structured up-titration, and individualized prioritization is summarized in Figure 1.

## Up-Titration Strategies and Common Pitfalls

Achieving target or maximally tolerated doses of GDMT is a key determinant of long-term outcomes in HFrEF. Nevertheless, real-world studies consistently demonstrate low rates of successful up-titration, often due to clinician hesitation, inadequate follow-up, or misattribution of symptoms to therapy-related adverse effects [8]. Key initiation criteria, contraindications, and monitoring parameters for GDMT in HFrEF are outlined in Table 2.

**Table 2:** Initiation Criteria, Contraindications, and Monitoring for GDMT in HFrEF

Therapy	Key initiation criteria	Major contraindications	Monitoring parameters
ARNI/ACEi/ARB	SBP ≥ 90–100 mm Hg; stable renal function	History of angioedema; pregnancy; severe hyperkalemia	BP, serum creatinine, potassium
β-blockers	Euvolemic, clinically stable patient	Cardiogenic shock; severe bradycardia; advanced AV block	HR, BP, symptoms of congestion
MRA	eGFR ≥ 30 mL/min/1.73 m^2^; K ≤ 5.0 mmol/L	Severe renal dysfunction; hyperkalemia	Potassium, renal function
SGLT2 inhibitors	Stable HFrEF with or without diabetes	Type 1 diabetes; active ketoacidosis	Renal function, volume status

HFrEF: heart failure with reduced ejection fraction; GDMT: guideline-directed medical therapy; ARNI: angiotensin receptor–neprilysin inhibitor; ACEi: angiotensin-converting enzyme inhibitor; ARB: angiotensin receptor blocker; MRA: mineralocorticoid receptor antagonist; SGLT2: sodium–glucose cotransporter 2; BP: blood pressure; HR: heart rate; SBP: systolic blood pressure; AV: atrioventricular.

### Principles of safe up-titration

Dose up-titration should be gradual, structured, and guided by frequent clinical assessment. Blood pressure, heart rate, renal function, and serum electrolytes should be monitored closely, particularly following initiation or dose escalation of renin–angiotensin system inhibitors and mineralocorticoid receptor antagonists [6]. Transient changes in renal function or mild hypotension are common during up-titration and should not automatically prompt therapy discontinuation. In many cases, adjustment of diuretic dose or temporary modification of titration speed allows continuation of disease-modifying therapy without compromising safety [9].

### Common pitfalls in clinical practice

Common barriers to successful up-titration include excessive concern regarding asymptomatic hypotension, underrecognition of congestion, and premature cessation of therapy in response to modest laboratory abnormalities. Polypharmacy and fragmented care further contribute to therapeutic inertia, particularly in older patients with multiple comorbidities [8]. Addressing these challenges requires a proactive, multidisciplinary approach, with clear treatment goals, patient education, and structured follow-up. When implemented effectively, systematic up-titration strategies substantially increase the proportion of patients receiving optimal doses of evidence-based therapies [10]. Table 3 provides a practical, implementation-oriented framework linking common clinical barriers to targeted strategies designed to facilitate safe and comprehensive GDMT optimization.

**Table 3:** Implementation Barriers to Optimal GDMT and Targeted Strategies for Resolution

Barrier category	Clinical scenario	Mechanistic concern	Targeted implementation strategy
Hemodynamic limitation	Borderline blood pressure	Risk of symptomatic hypotension	Prioritize SGLT2i and MRA; reduce non-prognostic vasodilators; adjust diuretics before down-titrating GDMT
Renal dysfunction	Rising creatinine after RAAS initiation	Fear of progressive kidney injury	Accept mild transient increase; reassess volume status; continue therapy with close monitoring
Hyperkalemia	Elevated potassium during RAAS/MRA therapy	Risk of arrhythmia	Dose adjustment; dietary counseling; potassium binders; avoid unnecessary discontinuation
Persistent congestion	Ongoing edema or dyspnea	Misattribution to GDMT intolerance	Optimize loop diuretics; confirm euvolemia before limiting disease-modifying therapy
Clinical inertia	Delay in initiating multiple therapies	Concern about polypharmacy or tolerability	Implement “four drugs in 4 weeks” strategy; protocol-driven low-dose initiation; follow-up within 1–2 weeks
Fragmented follow-up	Lack of structured reassessment	Failure to titrate toward target doses	Multidisciplinary HF clinics; nurse-led titration pathways; scheduled laboratory surveillance
Elderly or frail phenotype	Advanced age with comorbidities	Fear of intolerance or falls	Start low, titrate slowly; deprescribe non-essential drugs; shared decision-making

GDMT: guideline-directed medical therapy; MRA: mineralocorticoid receptor antagonist; SGLT2i: sodium–glucose cotransporter 2 inhibitor; RAAS: renin–angiotensin–aldosterone system.

## Management of Special Populations

Optimization of GDMT in HFrEF requires careful adaptation in specific patient populations, in whom standard treatment strategies may be limited by comorbidities, hemodynamic instability, or treatment intolerance. Individualized decision-making is essential to balance prognostic benefit against safety concerns [6, 7].

### Chronic kidney disease

Chronic kidney disease is highly prevalent among patients with HFrEF and is associated with worse clinical outcomes. Although concerns regarding renal dysfunction often lead to underutilization of disease-modifying therapies, substantial evidence supports the use of renin–angiotensin system inhibitors, mineralocorticoid receptor antagonists, and SGLT2 inhibitors in patients with mild to moderate renal impairment [5, 6]. Transient increases in serum creatinine following initiation of renin–angiotensin system inhibition are common and usually reflect hemodynamic changes rather than true renal injury. In most cases, therapy should be continued with close monitoring, as long-term renal and cardiovascular benefits outweigh short-term laboratory changes [9].

### Hypotension

Low blood pressure frequently complicates the management of HFrEF and represents a major barrier to GDMT optimization. Importantly, asymptomatic hypotension should not automatically prompt dose reduction or discontinuation of disease-modifying therapies. In such cases, reassessment of volume status and reduction of non-prognostic medications, including excessive diuretic dosing or vasodilators without mortality benefit, should be prioritized [10]. SGLT2 inhibitors and mineralocorticoid receptor antagonists are particularly valuable in hypotensive patients due to their minimal effects on systemic blood pressure [5, 9].

### Atrial fibrillation and bradyarrhythmias

Atrial fibrillation commonly coexists with HFrEF and complicates heart rate control and beta-blocker titration. In patients with uncontrolled ventricular rates, cautious up-titration of beta-blockers remains essential, while excessive bradycardia may necessitate dose adjustment or consideration of device therapy [7].

## Device Therapy and Advanced HFrEF

Although pharmacologic therapy remains the cornerstone of HFrEF management, device-based interventions provide substantial incremental benefit in appropriately selected patients. Device therapy should generally be considered after at least 3 months of optimized GDMT, allowing time for potential reverse remodeling and reassessment of left ventricular ejection fraction (LVEF) [6, 7].

### Implantable cardioverter-defibrillator (ICD)

ICD therapy is recommended for the primary prevention of sudden cardiac death in patients with the following: 1) LVEF ≤ 35%; 2) persistent systolic dysfunction despite ≥ 3 months of optimized GDMT; 3) New York Heart Association (NYHA) class II–III symptoms; and 4) reasonable life expectancy (> 1 year) with good functional status.

ICD therapy significantly reduces arrhythmic mortality but does not directly improve heart failure symptoms or reverse ventricular remodeling. Therefore, careful patient selection is essential, particularly in elderly or frail individuals where competing non-arrhythmic mortality may attenuate overall benefit. Shared decision-making remains central to appropriate implementation.

### Cardiac resynchronization therapy (CRT)

CRT improves ventricular synchrony, promotes reverse remodeling, reduces heart failure hospitalization, and improves survival in selected patients. CRT is recommended in individuals with the following: 1) LVEF ≤ 35%; 2) NYHA class II–IV symptoms despite optimized GDMT; 3) QRS duration ≥ 130 ms; and 4) left bundle branch block (LBBB) morphology.

The magnitude of benefit is greatest in patients with LBBB morphology and QRS duration ≥ 150 ms. Sinus rhythm is preferred; however, patients with atrial fibrillation may benefit when a strategy ensuring effective biventricular pacing is implemented. Device therapy should complement—not substitute—optimized GDMT.

**Figure 2:**
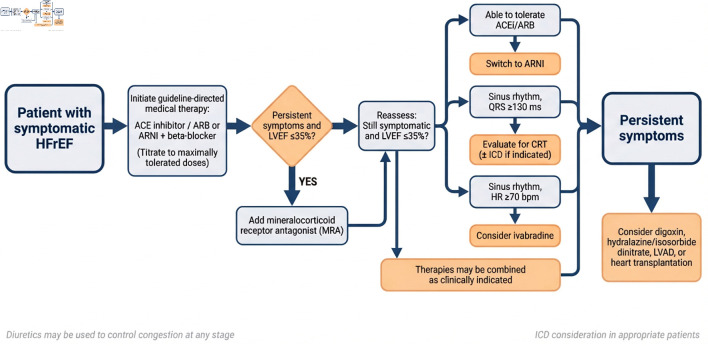
Stepwise treatment algorithm for patients with symptomatic heart failure with reduced ejection fraction (HFrEF). ACEi: angiotensin-converting enzyme inhibitor; ARB: angiotensin receptor blocker; ARNI: angiotensin receptor–neprilysin inhibitor; CRT: cardiac resynchronization therapy; HR: heart rate; ICD: implantable cardioverter-defibrillator; LVAD: left ventricular assist device; LVEF: left ventricular ejection fraction.

The integrated pharmacologic and device-based treatment pathway for symptomatic HFrEF, including criteria for ICD and CRT consideration, is summarized in Figure 2.

## Advanced Heart Failure and Referral Considerations

Despite comprehensive GDMT and appropriate device-based interventions, a subset of patients progress to advanced heart failure characterized by persistent symptoms, recurrent decompensation, and progressive end-organ dysfunction. Early identification of patients at risk for clinical deterioration is essential to facilitate timely referral to specialized heart failure centers.

### Referral red flags

Clinical features that should prompt consideration of referral for advanced heart failure evaluation include: 1) ≥ 2 heart failure hospitalizations within 12 months; 2) progressive renal dysfunction despite optimized therapy; 3) escalating diuretic requirements to maintain euvolemia; 4) symptomatic hypotension limiting further GDMT optimization; and 5) persistent NYHA class III–IV symptoms despite comprehensive therapy.

In addition to these clinical indicators, INTERMACS (Interagency Registry for Mechanically Assisted Circulatory Support) profiling provides a structured framework to stratify severity and trajectory of advanced disease [17]. Patients classified within INTERMACS profiles 4–7 often represent a critical window for referral before irreversible end-organ dysfunction develops. Profiles 1–3 reflect more advanced hemodynamic compromise and may necessitate urgent mechanical circulatory support.

Recognition of a declining clinical trajectory—rather than waiting for refractory cardiogenic shock—supports proactive referral strategies and may expand eligibility for durable mechanical circulatory support or heart transplantation. Delayed referral until end-stage disease may limit therapeutic options and adversely affect outcomes [7]. Comprehensive management of advanced HFrEF also includes structured non-pharmacologic interventions aimed at improving functional capacity and long-term stability.

## Role of Cardiac Rehabilitation in HFrEF

Comprehensive management of HFrEF extends beyond pharmacologic and device-based therapies. Structured cardiac rehabilitation, incorporating supervised exercise training, patient education, lifestyle modification, and psychosocial support, represents an essential but frequently underutilized component of contemporary heart failure care.

Randomized clinical trials and meta-analyses have demonstrated that exercise-based cardiac rehabilitation improves peak oxygen uptake, functional capacity, and health-related quality of life in patients with HFrEF. In addition, participation in structured rehabilitation programs has been associated with reductions in heart failure–related hospitalization, although mortality effects have been more variable across studies. Contemporary guidelines therefore recommend exercise training as a class I intervention in clinically stable patients with HFrEF [6, 7].

Beyond physiologic benefits, cardiac rehabilitation enhances self-management skills, medication adherence, dietary optimization, and early recognition of decompensation—factors that are central to successful implementation of GDMT. Importantly, referral rates to rehabilitation programs remain suboptimal, particularly among elderly patients, women, and socioeconomically disadvantaged populations.

Integration of cardiac rehabilitation into a multidisciplinary heart failure care pathway may improve long-term outcomes by complementing neurohormonal modulation with improvements in functional reserve, endothelial function, autonomic balance, and skeletal muscle metabolism. As with pharmacologic therapy, early referral following stabilization or hospitalization may maximize benefit. Thus, cardiac rehabilitation should be viewed not as an adjunctive afterthought, but as a foundational component of comprehensive HFrEF management.

## Follow-Up and Prognosis

Regular follow-up is a critical component of successful HFrEF management and is essential for ensuring sustained adherence, safe up-titration of therapy, and early identification of clinical deterioration. Patients undergoing initiation or escalation of GDMT require close monitoring of blood pressure, heart rate, renal function, and serum electrolytes, particularly during the first weeks of treatment [6, 10].

Long-term prognosis in HFrEF has improved substantially with contemporary GDMT; however, residual morbidity and mortality remain significant. Observational studies and clinical trials consistently demonstrate that patients receiving comprehensive, optimized GDMT experience lower rates of hospitalization and improved survival compared with those receiving partial therapy [8].

Importantly, evidence suggests that achievement of disease-modifying therapy, even at submaximal doses, confers meaningful prognostic benefit. This underscores the importance of early initiation, continued follow-up, and systematic optimization of therapy throughout the disease course [10].

## Contemporary Controversies and Evolving Perspectives in HFrEF Management

Despite the remarkable therapeutic advances achieved with contemporary GDMT, several areas of ongoing debate and uncertainty persist in the management of HFrEF. These controversies reflect the heterogeneity of the HFrEF population, limitations in trial representation, and the evolving understanding of ventricular remodeling biology.

### GDMT in elderly and frail patients

Older adults constitute an expanding proportion of the HFrEF population and frequently present with multimorbidity, polypharmacy, sarcopenia, cognitive impairment, and varying degrees of frailty [18]. Although landmark randomized trials consistently demonstrate preserved relative risk reduction across age strata, elderly and frail individuals remain underrepresented in pivotal studies, creating uncertainty regarding tolerability and real-world applicability.

Clinical hesitation in this population often stems from concerns about hypotension, renal dysfunction, electrolyte imbalance, falls, and drug–drug interactions. However, contemporary analyses suggest that while absolute rates of adverse effects may be higher, the proportional benefit of neurohormonal therapies remains substantial [9]. Frailty itself is an independent predictor of mortality and hospitalization, and therapeutic conservatism may inadvertently compound risk.

A nuanced strategy is therefore required—emphasizing low starting doses, slower titration, close laboratory surveillance, deprescribing of non-prognostic medications, and shared decision-making. Chronological age alone should not be considered a contraindication to foundational GDMT. Instead, individualized risk–benefit assessment should guide implementation.

### HFimpEF

The recognition of HFimpEF has introduced important conceptual and therapeutic questions. Increasing use of comprehensive GDMT has led to a growing subset of patients demonstrating reverse remodeling and meaningful improvement in LVEF. Whether this represents true myocardial recovery or pharmacologically maintained remission remains debated.

The TRED-HF trial demonstrated that withdrawal of neurohormonal therapy in patients with recovered dilated cardiomyopathy was associated with a high rate of relapse of ventricular dysfunction [19]. These findings suggest that apparent recovery frequently reflects disease control rather than cure. Consequently, discontinuation or down-titration of GDMT in HFimpEF may expose patients to renewed remodeling and clinical deterioration.

Current consensus therefore favors continuation of foundational therapies in most patients with HFimpEF, barring intolerance or contraindication. This phenotype highlights the dynamic and potentially reversible nature of ventricular remodeling, while simultaneously reinforcing the need for sustained neurohormonal modulation.

### Sex-based differences in HFrEF

Sex-related differences in heart failure epidemiology and therapeutic response remain incompletely characterized. Although HFrEF is more prevalent in men, women often present at older ages and with a greater burden of comorbidities. Importantly, women have historically been underrepresented in major heart failure trials, limiting the granularity of sex-specific analyses [20].

Available data suggest that women derive comparable relative benefit from foundational GDMT; however, differences in pharmacokinetics, body composition, autonomic tone, and blood pressure profiles may influence tolerability and dosing patterns. Emerging evidence also suggests potential variation in ventricular remodeling trajectories and arrhythmic risk. Recognition of sex-based disparities is essential to ensure equitable GDMT implementation and to avoid systematic undertreatment. Future prospective trials specifically powered for sex-based comparisons are needed to refine individualized therapeutic strategies.

### De-escalation strategies and residual risk despite quadruple therapy

The advent of quadruple foundational therapy has dramatically improved survival in HFrEF, yet substantial residual mortality and morbidity persist. Even under optimal implementation scenarios, modeling studies demonstrate that long-term risk remains considerable, reflecting ongoing myocardial injury, arrhythmogenesis, comorbid burden, and incomplete mechanistic targeting [21].

At the same time, increasing therapeutic complexity has prompted questions regarding de-escalation in clinically stable patients, particularly those with improved LVEF or borderline blood pressure. Current evidence does not support routine withdrawal of disease-modifying therapy in stable HFrEF, and premature de-escalation may reverse gains in ventricular remodeling. Rather than focusing on therapy reduction, contemporary management strategies emphasize sustained optimization, aggressive implementation of all evidence-based classes, identification of residual risk markers, and timely consideration of adjunctive pharmacologic, device-based, or advanced therapies.

## Key Clinical Take-Home Messages

Prioritize early, comprehensive initiation of all four foundational GDMT classes rather than prolonged stepwise sequencing. Adopt a practical “four drugs in four weeks” strategy, followed by structured, protocol-driven up-titration with close early follow-up. SGLT2 inhibitors confer early clinical benefit, reinforcing the importance of timely deployment during the vulnerable post-diagnosis phase. Most barriers to optimization (hypotension, renal dysfunction, hyperkalemia) are manageable and should not routinely lead to discontinuation of life-saving therapy. Despite quadruple therapy, residual risk persists—sustained optimization, phenotype-guided prioritization, and timely device or advanced HF referral are essential. Cardiac rehabilitation and multidisciplinary follow-up are integral components of comprehensive HFrEF management and support long-term implementation success.

## Data Availability

The authors declare that data supporting the findings of this study are available within the article.

## References

[1] Groenewegen A, Rutten FH, Mosterd A, Hoes AW (2020). Epidemiology of heart failure. Eur J Heart Fail.

[2] Savarese G, Lund LH (2017). Global public health burden of heart failure. Card Fail Rev.

[3] Packer M, Bristow MR, Cohn JN, Colucci WS, Fowler MB, Gilbert EM, Shusterman NH (1996). The effect of carvedilol on morbidity and mortality in patients with chronic heart failure. U.S. Carvedilol Heart Failure Study Group. N Engl J Med.

[4] McMurray JJ, Packer M, Desai AS, Gong J, Lefkowitz MP, Rizkala AR, Rouleau JL (2014). Angiotensin-neprilysin inhibition versus enalapril in heart failure. N Engl J Med.

[5] McMurray JJV, Solomon SD, Inzucchi SE, Kober L, Kosiborod MN, Martinez FA, Ponikowski P (2019). Dapagliflozin in patients with heart failure and reduced ejection fraction. N Engl J Med.

[6] McDonagh TA, Metra M, Adamo M, Gardner RS, Baumbach A, Bohm M, Burri H (2021). 2021 ESC Guidelines for the diagnosis and treatment of acute and chronic heart failure. Eur Heart J.

[7] Heidenreich PA, Bozkurt B, Aguilar D, Allen LA, Byun JJ, Colvin MM, Deswal A (2022). 2022 AHA/ACC/HFSA guideline for the management of heart failure: a report of the American College of Cardiology/American Heart Association Joint Committee on Clinical Practice Guidelines. Circulation.

[8] Greene SJ, Butler J, Albert NM, DeVore AD, Sharma PP, Duffy CI, Hill CL (2018). Medical therapy for heart failure with reduced ejection fraction: the CHAMP-HF registry. J Am Coll Cardiol.

[9] Tromp J, Ouwerkerk W, van Veldhuisen DJ, Hillege HL, Richards AM, van der Meer P, Anand IS (2022). A systematic review and network meta-analysis of pharmacological treatment of heart failure with reduced ejection fraction. JACC Heart Fail.

[10] Greene SJ, Fonarow GC, DeVore AD, Sharma PP, Vaduganathan M, Albert NM, Duffy CI (2019). Titration of medical therapy for heart failure with reduced ejection fraction. J Am Coll Cardiol.

[11] Mann DL (2015). Innate immunity and the failing heart: the cytokine hypothesis revisited. Circ Res.

[12] Zannad F, Rossignol P (2018). Cardiorenal syndrome revisited. Circulation.

[13] Neubauer S (2007). The failing heart—an engine out of fuel. N Engl J Med.

[14] Taqueti VR, Di Carli MF (2018). Coronary microvascular disease pathogenic mechanisms. Circulation.

[15] Felker GM, Lee KL, Bull DA, Redfield MM, Stevenson LW, Goldsmith SR, LeWinter MM (2011). Diuretic strategies in patients with acute decompensated heart failure. N Engl J Med.

[16] Digitalis Investigation Group (1997). The effect of digoxin on mortality and morbidity in patients with heart failure. N Engl J Med.

[17] Stevenson LW, Pagani FD, Young JB, Jessup M, Miller L, Kormos RL, Naftel DC (2009). INTERMACS profiles of advanced heart failure: the current picture. J Heart Lung Transplant.

[18] Forman DE, Maurer MS, Boyd C, Brindis R, Salive ME, Horne FM, Bell SP (2018). Multimorbidity in older adults with cardiovascular disease. J Am Coll Cardiol.

[19] Halliday BP, Wassall R, Lota AS, Khalique Z, Gregson J, Newsome S, Jackson R (2019). Withdrawal of pharmacological treatment for heart failure in patients with recovered dilated cardiomyopathy (TRED-HF): an open-label, pilot, randomised trial. Lancet.

[20] Lam CSP, Arnott C, Beale AL, Chandramouli C, Hilfiker-Kleiner D, Kaye DM, Ky B (2019). Sex differences in heart failure. Eur Heart J.

[21] Vaduganathan M, Claggett BL, Jhund PS, Cunningham JW, Ferreira JP, Zannad F (2020). Estimating lifetime benefits of comprehensive disease-modifying pharmacological therapies in patients with HFrEF. Circulation.

